# The impact of chronic disease on orphans’ quality of life living in extended social care services: a cross sectional analysis

**DOI:** 10.1186/s12955-016-0459-x

**Published:** 2016-04-05

**Authors:** Wadi B. Alonazi

**Affiliations:** Health Administration Department, King Saud University, P.O. Box 71115, Riyadh, 11587 Saudi Arabia

**Keywords:** Equity, Healthcare, Orphan patients, Quality of life, Quality of care, Tertiary care, Saudi Arabia

## Abstract

**Background:**

Owing to a scarcity of data or other causes, patient research on the orphan population is lacking in most societies. Consequently, the primary goal of this study was to explore quality of life (QOL) and quality of care (QOC) among orphan patients (OPs) receiving tertiary healthcare services in Saudi Arabia (SA).

**Method:**

This study used a cross-sectional, quantitative survey design. Participants included 216 OPs either currently undergoing or who had undergone treatment for common chronic diseases (CDs) (e.g. cardiovascular disease, cancer, stroke and arthritis) during the past 12 months. The survey utilised the brief form from the World Health Organization Quality of Life (WHOQOL-BREF) tool and evaluated healthcare access and effectiveness domains to scrutinise the socio-medical patterns of OPs based on their current medical episodes.

**Results:**

The descriptive analysis indicated that OPs’ overall QOL reached a moderate level (M = 3.90). Similarly, participants reported relatively high levels of healthcare access and treatment effectiveness (M = 4.14 and M = 4.29, respectively). Stroke patients reported the highest QOL score (M = 3.95), and groups of patients with other CDs reported greater access to healthcare and more effective treatment maintenance compared to the other groups (M = 4.19 and M = 4.43, respectively). Regression analysis was conducted to predict overall QOL based on perceived QOC, and access explained only 6.5 % of the variance. An analysis of variance showed significant differences only between OPs with cardiovascular disease and cancer (*P* = .001), with the former reporting better access to tertiary healthcare services than the latter.

**Conclusions:**

Although some CD patients reported relatively acceptable levels of access to healthcare and receipt of effective treatment, the improvement of OPs’ QOL and QOC poses a serious challenge for health policymakers.

## Background

Globally, individuals’ right to access healthcare services, obtain effective treatment, and live in complete wellbeing have been major challenges in the development of contemporary healthcare policies [1]. The Donabedian model for assessing quality offers a practical perspective that can be used to overcome this issue and increase the value of the healthcare system [2]. In this model, the structure of the healthcare system is associated with accessibility, and the process is measured in terms of effectiveness. Both accessibility and effectiveness are represented by quality of care (QOC) and the continuous evaluation of typical quality of life (QOL) outcomes [3]. For example, the Health-Related Quality of Life (HRQoL) instrument and the complete and brief forms of the World Health Organization Quality of Life measure (WHOQOL and WHOQOL-BREF, respectively) assess how health issues affect an individual’s wellbeing over time. Additionally, instruments such as the King’s Health Questionnaire (KHQ), the International Consultation on Incontinence Questionnaire-Short Form (ICIQ-SF), the Lung Cancer module (‬LC), and the Hospital Anxiety and Depression Scale (HADS) are utilised to measure the outcomes of specific diseases, disabilities, or disorders [4–7].‬‬‬‬‬‬‬‬‬‬‬‬‬‬‬‬‬‬‬‬‬‬‬‬‬‬‬‬‬‬‬‬‬‬‬‬‬‬‬‬‬‬‬

### Orphan patients in Saudi Arabia

The concept of patient-centeredness, which refers to an understanding of the implications of equity, can be employed to assist policymakers in achieving holistic social wellness goals and monitor healthcare system performance [8]. According to the health constitution (article one) of Saudi Arabia (SA), there are no disparities in healthcare system provision according to race, socioeconomic status, or any other group-related characteristic [9]. Orphan patients (OPs) receive comprehensive care in three stages. For early stage services, which commence at any age but do not extend past the age of 6 years, an orphan is monitored in nursery care services. Youth care services are provided to orphans between 6 and 18 years of age, and the mission of these services is to engage the orphans in normal social life activities. Orphans are then supervised by social care services and financially supported until the age of 33 years [10]. By definition, an orphan is younger than 18 years of age. However, based on the social care services criteria in SA, this study operationally defines an OP as a patient whose parents (either one or both parents) are unidentified and who receives tertiary healthcare services through the accredited social care services. According to UNICEF, there are approximately 150 million orphans worldwide, and Asia has the largest share (40 %) [11]. Underdeveloped countries, especially Arabic countries, have relatively more orphans than other nations; however, resources designed to address orphans’ emotional distress may be more frequently available in developed countries than in less developed countries [12]. Although orphans represent a minority population, few studies have addressed their physical, mental, social, or environmental wellbeing in the context of tertiary healthcare services [13]. Therefore, the overarching aim of this study was to measure the impact of certain chronic diseases (CDs) on orphans’ QOC and to explore how wellbeing domains were promoted in extended orphan-hood social care services.

### Orphan patients’ quality of life

Worldwide, the orphan population is both the most underserved segment and the most vulnerable to diseases. Due to rapid socioeconomic changes, children have lost one or both parents and even siblings; however, the increase in the number of orphans has remained an unaddressed issue in Saudi Arabian society. Because hundreds of children no longer have guardians, the government of SA established several homes that raise orphans with governmental protection until they reach the age of 18 years. The government of SA has implemented comprehensive social initiatives to integrate orphans into society, and many orphans share residences operated by social care organisations [14]. Subsequently, other philanthropic governmental organisations gradually incorporate orphans into social life, a process that normally occurs between the ages of 18 and 33 years. Therefore, a central mission of social care organisations is increasing social equity by raising and educating orphans and offering rehabilitation programs to facilitate their integration into society [15].

Although the concept of QOL has been thoroughly investigated in studies on individuals who have experienced medical episodes, little research has been conducted with ethnic groups or other specific populations. In a systematic review of research on children’s general wellbeing, Kumar and colleagues concluded that children with a high socioeconomic status were more likely to sustain a high QOL [16]. Additionally, in a South African study of children receiving HIV services at two community hospitals and 34 primary healthcare facilities, researchers found that OPs were more likely to suffer from mental disorders than non-OPs because they consistently faced barriers to accessing mental healthcare [17]. Similarly, many health-related studies have clearly identified the need to improve orphans’ reported QOL dimensions, especially those related to psychosocial factors [18, 19].

The segment of this population that may require the most substantial attention is the group that receives the least amount of care. Although OPs may suffer from a lack of sufficient social support, their medical needs including, access to healthcare services and effective treatments, should not be neglected within the healthcare system [20].

### Quality of care

The Institute of Medicine (IOM) defined QOC as a baseline measurement of on-going efforts to achieve desired health outcomes through assessment and improvement [21]. Again, the WHO highlighted that major characteristics of a sustainable healthcare system include the delivery of timely, reasonable, and skilful healthcare as well as adherence to contemporary knowledge and practices [22, 23].

In a cross-sectional survey of 707 orphans in Kenya, income predicted only a portion of the improvement in healthcare access. Additionally, the survey reported that empowering community-based programs and skilled clinicians are needed and are essential for improving OPs’ overall health status outcomes [24]. As previously mentioned, orphans are more likely to face challenges in both attaining comprehensive mental health services and accessing healthcare [17].

According to WHO statistics [25], the major causes of mortality are cardiovascular diseases, CDs, specific non-CDs, cancers, and injuries (46, 14, 13, 10, and 9 %, respectively). In 2002, over 69 % of the deaths in SA were attributed to CDs. This statistic is derived from published reports based on the general population; however, there is a paucity of information on minority groups or recipients of tertiary medical services [26–28]. The study of the histories of patients with CDs and other social risk factors provides a unique opportunity to explore factors associated with decreased health costs [29]. Actions taken to prevent major CDs should focus on controlling key risk factors through well-integrated health policies. For example, Al-saadi et al. recommended the early diagnosis of diseases, especially arthritis, among adolescents in SA [30].

A recent study conducted by Al-Jobair and colleagues highlighted that OPs must access an appropriate amount of healthcare services, particularly dental services, because the majority of medical services they currently receive are emergency services [31]. Orphans’ lack of access to essential dental services demonstrates these patients’ limited access to routine and tertiary services relative to emergency services. Literature reviews have suggested that to reduce CDs, it is necessary to maintain public health promotion campaigns, promote healthy activities, and introduce effective medical treatments and interventions [15, 31, 32].

In conclusion, many health-related studies have measured QOL and the outcomes of receiving medical services separately, but the impact of health services on OPs in SA has not yet been explored. The need to investigate such relationships is increasing, especially in the context of tertiary medical episodes [19].

## Methods

### Study design

This quantitative study employed a cross-sectional design and was conducted from January to June 2015. It included the WHOQL-BREF and factors related to QOC. The WHOQL-BREF measures physical, mental, social, and environmental wellbeing by assessing individuals’ perceptions in the context of their culture and value systems, personal goals, standards and concerns. The tool is reliable and robust for use in SA. The overall goal of this study was to explore the QOL and QOC of OPs receiving tertiary healthcare services in SA.

### Study setting

Officially, 12 social care organisations in SA provide social services to approximately 20,000 male and female OPs. This study was conducted at one organisation in Riyadh, where approximately 2123 orphans were registered. This social care organisation was selected primarily because it is the only organisation in the cosmopolitan area that provides tertiary health care services. The minimum and maximum ages for registration are 18 and 33 years, respectively.

### Sample

The study sample included only OPs who were currently receiving or had previously received tertiary healthcare services within the past 12 months. Within the sample, the primary diagnoses included cardiovascular diseases, cancers, stroke, arthritis, and other CDs. According to the referral forms provided by the orphan social organisation coordinators, approximately 523 OPs received regular treatment through tertiary healthcare services.

### Procedure

Ethical approval was obtained from the orphan social care organisation. Prior to data collection, liaisons from the organisation received training on the study objectives and data collection procedures. Following OPs’ routine inquiry with receptionists, liaisons approached the OPs in a friendly manner. Participants who agreed to participate were provided with a sealed envelope that contained the study questionnaire. Participants were informed that they should return questionnaires to the designated boxes in the reception area within 1 week. All participants were informed (both in writing and verbally) that study participation was voluntary and would not affect the services that they received. In addition, participants were not asked to disclose their names.

### Study instruments

In addition to gathering information about basic demographic characteristics, we utilised the WHOQOL-BREF to assess QOL and two QOC domains: access and effectiveness. Participants evaluated QOC performance in terms of organisational access as well as hospital amenities and clinical factors on a 5-point Likert scale.

## Results

A total of 216 orphans aged 20–33 years participated in this study. As shown in Table 1, the sample consisted of 12 employed individuals (5.6 %) and 204 unemployed individuals (94.4 %). Approximately half of the unemployed participants (49 %) were between the ages of 20 and 30 years.

**Table 1:** Orphan patients’ socio-demographic characteristics

Marital status	Employed (*n* = 12)				Unemployed (*n* = 204)			
	Respondent’s age				Respondent’s age			
	≤20 years	21–30	31–33	%	≤20 years	21–30	31–33	%
Married	0	1	0	8.3	4	14	3	10.3
Single	4	5	0	75.0	75	99	5	87.7
Divorced	0	1	1	16.7	0	4	0	2.0
Total	4	7	1	100.	79	117	8	100

As shown in Table 2, slightly more than half of the respondents (*n* = 112; 51.9 %) were male, and the remainder were female (*n* = 104; 48.1 %). Slightly less than half of the participants had no formal education (*n* = 105; 49 %), 87 subjects (40 %) completed a high school certificate or below and the remainder obtained a bachelor’s degree (*n* = 24; 11 %). The majority of patients were diagnosed with cardiovascular disease (*n* = 128; 59.3 %); approximately one-quarter were diagnosed with cancer (*n* = 57; 26.4 %); and substantially fewer were diagnosed with stroke (*n* = 16; 7.4 %), arthritis (*n* = 12; 5.6 %), or other CDs (*n* = 3; 1.4 %).

**Table 2:** Gender-based comparisons of education level and chronic disease type

Type of CD	No Education (*n* = 105)			High school and below (*n* = 87)			Bachelor degree (*n* = 24)			Overall (*N* = 216)	
	M	F	%	M	F	%	M	F	%	Total	%
Cardiovascular	22	41	60	16	35	59	6	8	58	128	59.3
Cancer	22	5	25.7	17	8	29	3	2	21	57	26.4
Stroke	4	3	6.7	4	2	6.9	3	0	12.5	16	7.4
Arthritis	6	0	5.7	4	0	4	2	0	8.5	12	5.6
Others	2	0	1.9	1	0	1.1		0	0.0	3	1.4

*Abbreviations*: *CD* chronic disease, *F* female, *M* male

The mean (M) scores on the QOL and QOC domains, by gender, are presented for each group in Table 3. In terms of QOL, arthritis patients exhibited the highest mean score, i.e., 3.95, and patients diagnosed with other CDs displayed the lowest score, i.e., 3.80. The results also showed that patients with other CDs accessed hospitals more frequently (M = 4.19) than the other groups and that cardiovascular patients reported the least frequent access (M = 4.08). Patients with other CDs reported receiving the most effective treatment (M = 4.43), whereas those with arthritis reported receiving the least effective treatment (M = 4.15).

**Table 3:** Means for chronic disease type based on gender-related health patterns and health domains

Overall Domain	Cardiovascular		Cancer		Stroke		Arthritis	Other	Overall Groups
	M	F	M	F	M	F	M	M	
Access	4.17	3.99	4.2	4.1	4.28	3.99	4.1	4.19	4.14
Overall means	4.08		4.14		4.13		4.14	4.19	
Effectiveness	4.39	4.15	4.5	4.17	4.63	3.90	4.15	4.43	4.29
Overall means	4.27		4.34		4.27		4.15	4.43	
QOL	3.97	3.90	3.95	3.86	3.96	3.90	3.92	3.8	3.90
Overall means	3.94		3.91		3.95		3.92	3.8	

*Abbreviations*: *F* female, *M* male, *QOL* quality of life

To establish the relationship between overall QOL, access, and effectiveness, we performed multiple regression analysis (see Table 4). The results showed a negative association between amenity quality and QOL. Specifically, holding all other variables constant, we found that for each unit increase in the quality of amenities, overall QOL decreased 0.062 units. Therefore, based on Table 4, we developed the following overall regression equation for QOL:

**Table 4:** Multiple regression analysis for prediction of overall quality of life

Model	Unstandardized coefficients		Standardized coefficients	t	*p*-value*
	B	Std. error	Beta		
(Constant)	3.624	0.296		12.25	0.000
Access-Tertiary Criteria	0.065	0.051	0.089	1.271	0.205
Access-Organizational Criteria	0.037	0.050	0.05	0.726	0.469
Quality-Amenities	−0.062	0.025	−0.174	−2.523	0.012
Quality-Clinical	0.044	0.037	0.081	1.171	0.243

*P < 0.05

$$ \begin{array}{l} Overall\ QOL= 3.624 + 0.065\;*\; Access\mathit{\hbox{-}} Tertiary\  Criteria + 0.037\ * Access\mathit{\hbox{-}} Organisational\  criteria\mathit{\hbox{--}}\  0.062\ *\\ {} Quality\mathit{\hbox{-}} Amenities + 0.044\ * Quality\mathit{\hbox{-}} Clinical\end{array} $$

To identify the perceived differences in accessing and obtaining effective treatment between the respondents with different CDs, we performed an analysis of variance (ANOVA) to compare the OPs’ overall access to tertiary healthcare. The analysis indicated that of all domains, only access to tertiary healthcare services showed a significant difference. The results of the analysis are presented in Table 5. The results suggest that only the cardiovascular disease and cancer groups exhibited significant between-group differences. Specifically, cancer patients reported significantly greater perceived access to tertiary healthcare services than those with cardiovascular diseases (*P* = 0.001).

**Table 5:** The effects of chronic disease on accessing healthcare among orphan patient groups

Dependent variable	CD type	Mean difference	Std. error	*p*-value*	95 % Confidence interval	
Access to Tertiary Services	Cancer	−.23019^*^	.05618	.001	−.38	−.07
	Stroke	−.15234	.09355	.481	−.41	.11
	Arthritis	−.21484	.10651	.261	−.51	.08
	Others	.07682	.20605	.996	−.49	.64

*Abbreviations*: *CD* chronic disease

*P < 0.05

## Discussion

The study findings revealed a moderate level of QOL and a high level of QOC among OPs receiving tertiary healthcare services in the Riyadh region. Although unidentified-parent orphans may not typically be provided with the emotional support that they need from biological parents, the results suggest that their wellbeing can be increased through interactions with others, such as peers and social workers [9]. As previously discussed, the development of a CD may influence OPs’ healthcare outcomes. Although medicine has significantly improved, OPs are more vulnerable to social and medical distress than any other segment of the population [33].

Unexpectedly, participants in the 21–30 age group were frequently diagnosed with arthritis. Physical inactivity is associated with a high risk of developing other CDs, such as cardiovascular diseases and obesity. Although disabilities associated with arthritis are projected to increase beginning at age 15 in SA, globally, the typical age of disease onset is 45 years [34]. In SA, this prevalence may be due to the absence of early screening [30].

Clearly, some OPs undervalued broad hospital characteristics, including amenities. Indeed, the quality of amenities was negatively correlated with QOL. Because clinical quality was an overriding concern, amenities were less critical [35]. This result suggests that domains that are not associated with patient-centredness are less important to OPs undergoing tertiary healthcare.

This study has several limitations. For example, the medical episodes examined in this study’s exploration of the central domains related to OPs’ QOL and QOC addressed the five most common CDs in SA. Research focusing on a single disease would likely provide valuable insight.

One implication of this study is that it is necessary to focus more attention on clinical factors and less on hospital amenities. Public health policymakers face the new challenge of crossing the social chasm between OPs and society to increase OPs’ wellness.

## Conclusions

While there were some variations, the overall results indicated that the patients were able to access healthcare services, and maintain effective treatment. Specifically, the findings revealed a moderate level of QOL and a high level of QOC among OPs receiving tertiary healthcare services. Furthermore, the results indicated that QOC had a minimal, but significant, effect on QOL. In conclusion, public health policymakers should focus more attention on clinical factors and less on hospital amenities.

## Abbreviations

ANOVA: analysis of variance
CD: chronic disease
HADS: Hospital Anxiety and Depression Scale
HRQoL: Health-Related Quality of Life scale
ICIQ-SF: International Consultation on Incontinence-Short Form
KHQ: King’s Health Questionnaire
LC: lung cancer module
OPs: orphan patients
QOC: quality of care
QOL: quality of life
SA: Saudi Arabia
WHOQOL: World Health Organization Quality of Life measure, complete form
WHOQOL-BREF: World Health Organization Quality of Life measure, brief form

## References

[1] Brody DS (1980). The Patient’s role in clinical decision-making. Ann Intern Med.

[2] Donabedian A (1988). The quality of care. How can it be assessed?. JAMA.

[3] Stevens AJ, Longson C (2013). At the center of health care policy making: the use of health technology assessment at NICE. Med Decis Making.

[4] Khullar V (2012). Patient-reported outcomes and different approaches to urinary parameters in overactive bladder: what should we measure?. Int Urogynecol J.

[5] Tamanini JTN, Dambros M, D’Ancona CAL, Palma PCR, Netto R (2004). Validation of the"international consultation on incontinence questionnaire-short form"(ICIQ-SF) for Portuguese. Rev Saude Publica.

[6] Moinpour CM, Lovato LC, Yee M, Blumenstein BA, Savage MJ, Troxel A, Eisenberger M, Veith RW, Higgins B, Skeel R. Quality of life in advanced prostate cancer: results of a randomized therapeutic trial. J Natl Cancer Inst. 1998;90(20):1537–44. 10.1093/jnci/90.20.1537 9790546

[7] Snaith RP (2003). The hospital anxiety and depression scale. Health Qual Life Outcomes.

[8] Saha S, Beach MC, Cooper LA (2008). Patient centeredness, cultural competence and healthcare quality. J Natl Med Assoc.

[9] Mobaraki A, Soderfeldt B (2010). Gender inequity in Saudi Arabia and its role in public health. East Mediterr Health J.

[10] Ashaalan L, Al-zeiby I (2015). Methods of care for children living in orphanages in Saudi Arabia. J Int Educ Res.

[11] Orphans:Press centre. UNICEF. Available from: http://www.unicef.org/media/media_45279.html. Accessed 30 June 2015.

[12] Bodenheimer T, Lorig K, Holman H, Grumbach K (2002). Patient self-management of chronic disease in primary care. JAMA.

[13] Aljaman S, Aljaman S, Hamoud A (2012). Orphan problems inside and outside the country. Albasrah Res J.

[14] Ministry of Social Affairs (MOSA). Annual Statistics Report. Availabe from: http://www.mosa.gov.sa/sites/default/files/uploads/Statistical%20Year%20book.pdf. Accessed 3 Jan 2016.

[15] Williams SD, Hansen K, Smithey M, Burnley J, Koplitz M, Koyama K, Young J, Bakos A. Using social determinants of health to link health workforce diversity, care quality and access, and health disparities to achieve health equity in nursing. Public Health Rep. 2014;129 Suppl 2:32–6. 10.1177/00333549141291S207 PMC3863698 24385662

[16] Kumar S, Kroon J, Lalloo R (2014). A systematic review of the impact of parental socio-economic status and home environment characteristics on children’s oral health related quality of life. Health Qual Life Outcomes.

[17] Pearcey PA (1995). Achieving research‐based nursing practice. J Adv Nurs.

[18] Al Robaee AA (2007). Assessment of quality of life in Saudi patients with vitiligo in a medical school in qassim province, Saudi Arabia. Saudi Med J.

[19] Alonazi W, Thomas S (2014). Quality of care and quality of life: convergence or divergence?. Health Serv Insights.

[20] Benatar SR (2004). Health care reform and the crisis of HIV and AIDS in south Africa. N Engl J Med.

[21] Corrigan JM, Donaldson MS, Kohn LT (2001). Crossing the quality chasm: a new health system for the 21st century.

[22] Skevington SM, Lotfy M, O’Connell KA (2004). The world health Organization’s WHOQOL-BREF quality of life assessment: psychometric properties and results of the international field trial. A report from the WHOQOL group. Qual Life Res.

[23] DeVoe J (2008). The unsustainable US health care system: a blueprint for change. Ann Fam Med.

[24] MacGuire JM (2006). Putting nursing research findings into practice: research utilization as an aspect of the management of change. J Adv Nurs.

[25] World Health Organization (WHO). Global Health Observatory (GHO) data. Available from: http://www.who.int/gho/en/. Accessed 8 Oct 2015.

[26] Norekval TM, Wahl AK, Fridlund B, Nordrehaug JE, Wentzel-Larsen T, Hanestad BR (2007). Quality of life in female myocardial infarction survivors: a comparative study with a randomly selected general female population cohort. Health Qual Life Outcomes.

[27] United Nations Children’s Emergency Fund (UNICEF). Promoting quality education for orphans and vulnerable children: A sourcebook of programme experiences in Eastern and Southern Africa. Availabe from: http://www.unicef.org/french/education/files/Promoting_Quality_Education_for_Orphans_and_Vulnerable_Children_Programmes_from_Eastern_and_Southern_Africa.pdf. Accessed 5 July 2015.

[28] Williamson J, Greenberg A (2010). Families, not orphanages.

[29] Martin LR, Williams SL, Haskard KB, Dimatteo MR (2005). The challenge of patient adherence. Ther Clin Risk Manag.

[30] Al-saadi MM, Al Zamil FA, Bokhary NA, Al Shamsan LA, Al Alola SA, Al Eissa YS (2009). Acute septic arthritis in children. Pediatr Int.

[31] Al-jobair AM, Al-Sadhan SA, Al-Faifi AA, Andijani RI (2013). Medical and dental health status of orphan children in central Saudi Arabia. Saudi Med J.

[32] Bauer UE, Briss PA, Goodman RA, Bowman BA (2014). Prevention of chronic disease in the 21st century: elimination of the leading preventable causes of premature death and disability in the USA. Lancet.

[33] McCalman J (2009). Silent witnesses: child health and well-being in England and Australia and the health transition 1870–1940. Health Sociol Rev.

[34] Badley EM, Wang PP (1998). Arthritis and the aging population: projections of arthritis prevalence in Canada 1991 to 2031. J Rheumatol.

[35] Goldman DP, Vaiana M, Romley JA (2010). The emerging importance of patient amenities in hospital care. N Engl J Med.

